# Differences over 12 Years in Food Portion Size and Association with Excess Body Weight in the City of São Paulo, Brazil

**DOI:** 10.3390/nu10060696

**Published:** 2018-05-30

**Authors:** Jaqueline Lopes Pereira, Paula Victória Félix, Josiemer Mattei, Regina Mara Fisberg

**Affiliations:** 1 Department of Nutrition, School of Public Health, University of São Paulo, São Paulo SP 01246-904, Brazil; jaque.lps@gmail.com (J.L.P.); paula.victoria@gmail.com (P.V.F.); 2 Department of Nutrition, Harvard T. H. Chan School of Public Health, Boston, MA 02115, USA; jmattei@hsph.harvard.edu

**Keywords:** portion size, eating frequency, food intake, energy contribution, diet, obesity, overweight

## Abstract

Although changes in Brazilian diet have occurred over the last decades, there is no evidence about differences in food portion sizes (FPS) over time. Therefore, we aimed to evaluate the association of FPS with excess body weight (EBW), and to monitor differences in the population from São Paulo, Brazil, from 2003 to 2015. Data came from three cross-sectional population-based studies with 5270 individuals aged ≥12 years in 2003, 2008, and 2015. Dietary data were obtained from 24-h recalls. Logistic regression models were used to evaluate the association between FPS and EBW. Over the years, there was a diverse variation in FPS, with an increase in some groups (white meat, salted snacks, coffee/tea, eggs) and decrease in others (rice, red meat, sweets, pasta, sandwiches, cold cuts). The percentage of people reporting the intake of six food groupings (rice, white meat, sweets, fruits, commercial juices, toasts/biscuits) increased in the period. In this population, EBW was associated with larger FPS of 11 of the 30 food groupings investigated (cold cuts, fried snacks, fruit and commercial juices, pizza, red meat, rice, salted snacks, soft drinks, soups, sugar). These findings could support future interventions and policies for optimal food intake in Brazil.

## 1. Introduction

In recent years, the portion size of foods (especially energy-dense foods) has been intensively investigated as a possible contributor to the rise in prevalence of excess body weight (EBW, including both overweight and obesity) in diverse populations [1,2]. The connection between EBW and portion size has often been linked to rising portion sizes of commercialized foods [3,4,5] and amount consumed per eating occasion [6]. In addition, experimental studies in humans have demonstrated that exposure to larger portion sizes for successive days resulted in higher energy intake [7,8,9,10], since individuals tend to not compensate the extra calories from larger portion sizes by eating less in the subsequent meals, which may influence body weight status. 

However, population studies in different countries have observed inconsistent trends in portion sizes over the years, with portion sizes of some foods decreasing, while others were increasing [11,12,13,14,15]. Additionally, few studies have demonstrated the link between EBW and larger portion sizes in free-living populations [16,17,18]. In Brazil, where more than half of the population have EBW, and one in every five adults is obese [19], only two studies have investigated the association between food portion sizes and EBW. One study with the population of São Paulo in 2008, found a positive association between EBW and larger portion sizes of pizza, red meat, rice, salted snacks, and soft drinks [20]. The other study, investigating the portion sizes of beverages in a representative sample of the country, found a positive association between alcoholic beverages and soft drinks and excess body weight [21]. 

Although changes in Brazilian dietary patterns have occurred over the last decades in parallel with the rise in the prevalence of EBW [22,23], as far as we know, there is no evidence about differences on food portion sizes (which are defined as the weight of food or beverage in grams consumed per eating occasion) over the time. Therefore, the aim of this study was to evaluate the association of food portion sizes with body weight status, and to monitor the differences over the years in a representative population from the city of São Paulo, Brazil, from 2003 to 2015.

## 2. Materials and Methods 

### 2.1. Study Design and Population

Data were collected from three cross-sectional samples of individuals from São Paulo city, Southeastern Brazil, who were interviewed in the Health Survey of São Paulo (ISA-Capital) conducted in 2003, 2008 and 2015. ISA-Capital is a population-based study with a probability sample of individuals living in permanent households located in the urban area of São Paulo, with sampling stratified by clusters carried out in two stages to ensure representativeness at population level: urban census tracts and households. Details of the 2003, 2008 and 2015 surveys, including the sampling design and the sample size calculation, were previously published [24,25,26]. 

The samples of the present study included participants residing in São Paulo, aged 12 years or older, of both sexes, and, in the case of women, not being pregnant or lactating during data collection. A sub-sample of the ISA-Capital was drawn to compose the “Health Survey of São Paulo with Focus in Nutrition Study” (ISA-Nutrition). Nutritional information was collected from 2398 individuals from 2003 ISA-Nutrition, 1662 individuals from 2008 ISA-Nutrition, and 1742 from 2015 ISA-Nutrition. Individuals with incomplete anthropometric data required for body mass index (BMI) calculation were excluded from the analysis, resulting in 2100, 1586 and 1689 individuals respectively. In order to ensure data quality, the extreme values of energy intake (below the 1st percentile and above the 99th percentile by sex) were excluded, resulting in 2060, 1556 and 1654 individuals per respective cycle. The final sample for this study comprised 5270 participants: 1711 adolescents, 1865 adults and 1143 older adults.

This survey was approved by the Ethics Committee on Research of the School of Public Health, University of São Paulo (reference number # 56958916.5.0000.5421). Written informed consent/assent was obtained from all participants before commencement of the study.

### 2.2. Data Collection

Demographic, socioeconomic, and lifestyle data were collected from households using a structured questionnaire administered by trained interviewers. The following variables were used for the present study: age, categorized in age groups (adolescents—from 12 to 19 years old, adults—from 20 to 59 years old, and older adults—aged 60 years or more); sex (male or female); self-reported skin color (white or non-white); per capita family income (calculated by summing the monetary income reported by all family members and dividing by the number of family members, the values were classified as ≤1 minimum wage, or >1 minimum wage according to the survey year); and householder education level (measured in years of schooling and categorized as ≤9 (completed elementary school) or >9 years of study (high school or more) and considering householder as the person responsible for the family or head of the family). Participants were classified according to their smoking status as current, former, or never smoker. Alcohol consumption was classified as ≤3 times per week, >3 times per week, or never. Global physical activity level was obtained from the International Physical Activity Questionnaire—IPAQ long version [27] which was validated for the Brazilian population [28], and participants were classified as meeting or not meeting the recommendation according to World Health Organization (WHO) guidelines [29].

Self-reported height and weight were used to calculate the BMI (BMI = weight (kg)/height (m)^2^), which was used to classify the individuals according to body weight status as without vs. with excess body weight as follows (1) adolescents: BMI < p85 vs. BMI ≥ p85 [30]; (2) adults: BMI < 25 kg/m^2^ vs. BMI ≥ 25 kg/m^2^ [31]; (3) older adults: BMI < 28 kg/m^2^ vs. BMI ≥ 28 kg/m^2^ [32].

Food consumption information was obtained from a 24-h dietary recall (24HR) collected by trained interviewers, representing all days of the week, and season of the year, using the procedures of the Multiple Pass Method (MPM). The respondent, in this process, is guided through five sequential steps: quick list, forgotten foods list, meal time and eating occasions, detail cycle (which includes portion sizes consumed) and final probe. This standardized process maintains the attention of the respondents, which helps them recall all items consumed and reduces respondent burden [33]. Participants were instructed to inform amounts of foods and beverages in household measures and describe them as detailed as possible, including eating occasions, meal time, cooking methods, seasonings and brand names. To guide the estimation of the portion sizes, manuals with photos of household measures were provided to individuals. In specific situations, where the participant could not report the amount consumed, e.g., amount of salt and oil used for salad seasoning, a standard portion was included. During all data collection, quality control of the 24HR was conducted in order to identify and correct possible reporting errors.

After dietary data collection, all household measures reported in each 24HR were converted into grams and milliliters according to Brazilian publications [34,35]. Food consumption data were entered in the Nutrition Data System for Research (NDSR) software (version 2014), developed by the Nutrition Coordinating Center, University of Minnesota, Minneapolis, MN, United States of America, which uses the food composition table developed by the United States Department of Agriculture as main data source. The nutritional values of the foods present in the program were compared with the nutritional values of foods available in Brazilian national tables [36]. A consistency analysis of dietary data was performed to identify and correct possible errors in processing and data collection.

### 2.3. Food Grouping

Around 1400 different foods and preparations were reported in all 24HR, which were classified into 46 food groupings, in order to determine those that mostly contributed to the total energy intake of residents in São Paulo city. The food grouping was based on the frequency of consumption, nutritional value and dietary habits of São Paulo population. Foods and mixed dishes (such as feijoada, yakssoba and risotto) consumed by less than 5% of the population were not organized for grouping due to the low prevalence and high variability of nutritional values. Details of food grouping are reported in a previous ISA-Nutrition study by Pereira et al. [20] and the list of foods that were included in each food grouping is described in Table S1.

**Figure 1:**
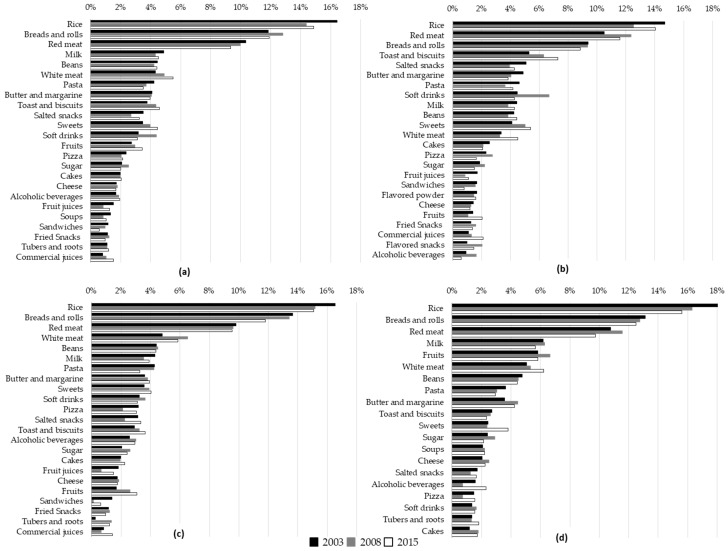
Percentage of energy contribution (%) of food groupings consumed by the population of the ISA-Nutrition in 2003, 2008, and 2015. (**a**) Description of total population; (**b**) Description of adolescents; (**c**) Description of adults; (**d**) Description of older adults. The list of foods included in each food grouping is described in Table S1.

For portion size analysis, we considered food groupings that contributed up to 90% of total energy intake using the method proposed by Block et al [37], additionally to those consumed by at least 10% of the population, resulting in 31 food groupings analyzed, as described in the results section (Figure 1).

### 2.4. Portion Size, Energy Density, and Energy Intake/Estimated Energy Requirement Ratio 

Portion size was defined as total amount of food or beverage in grams that a person consumed at a particular meal occasion [38]. It was estimated by the total intake (in grams) of the items included in the food grouping, divided by the number of eating occasions in which the food grouping was consumed by each individual. For example, if the participant reported the intake of one medium ladle of brown beans for lunch and one medium ladle of brown beans for dinner, her/his portion size is 86 g. However, if the participant reported the intake of two medium ladles of brown beans for lunch and did not consume beans in any other meal, her/his portion size is 172 g. As the intention of this study is to show changes in the average portion size for those who consume a specific item, only individuals who consumed a certain food grouping were included in the analysis of this group.

Energy density (defined as the amount of energy per gram of food) was estimated for each individual dividing the daily energy intake reported on the 24HR (EI, in kcal) by the total amount consumed, in grams. EI of each 24HR was estimated according to Food and Agriculture Organization of the United Nations (FAO) methodology [39], which determines energy conversion coefficients of nutrients more accurately than other traditional methods. It takes into account the digestion and absorption of nutrients, reflecting the amount of energy that can actually be used by the human organism and considered in equation energy balance. Besides considering the energy value attributable to alcohol consumption, this method also distinguishes the energy value of available carbohydrate (starch polysaccharides), which would be 4 kcal/g, to available energy of fibers, which would be 2 kcal/g, which assumes a mixture of fermentable (70% of total) and non-fermentable fiber.

Energy Intake/Estimated Energy Requirement ratio (EI/EER) was used as a continuous measure in the models investigating the association between EBW and food portion sizes, in order to statistically adjust for energy intake misreporting. A ratio higher than one means overreporting of energy intake, and a ratio lower than one means underreporting. The magnitude of the ratio reflects the difference in the error of energy reporting across the individuals. This method avoids the loss of statistical power that occurs when applying cut-off points to identify under or over-reporters [40]. EER were calculated using the formulas of the Institute of Medicine of the National Academies [41].

### 2.5. Statistical Analysis 

Data were analyzed with Stata software version 13.0 (StataCorp, College Station, TX, USA) in survey mode, considering the sample design and weights for population representativeness. General and dietary variables were described using absolute and relative frequencies. Differences between the variables by BMI category (without EBW versus with EBW) were tested according to Pearson’s chi-square test for categorical variables, and Mann–Whitney tests for continuous non-parametric variables. Unregistered variables were encoded as missing. Descriptive statistics were used to describe prevalence of consumers, mean, median of the portion sizes (in g), and standard error of each food grouping for consumers only.

The association between food portion sizes (continuous variable) and EBW (dependent variable), were evaluated by stepwise forward logistic regression models controlling for confounding factors. The models were adjusted for dietary energy density (kcal/g), age (years), gender (male or female), racial self-identification (white or non-white), household income (≤1 or >1 minimal wage per person in the family), householder education (≤9 or >9 years), smoking status (never, former, or current smoker), alcoholic beverages intake (never, ≤3 times per week, or >3 times per week), physical activity level (meet or do not meet WHO recommendation), Energy intake/Estimated Energy Requirement ratio, day of the week (weekday or weekend), intention to lose weight (yes or no), number of eating occasions, and survey year (2003, 2008, or 2015). The model for beans was additional adjusted for intake of rice according to the procedure of Mattei et al. [42], due to high correlation (*r* = 0.6434 *p* < 0.0001) between these two foods in this Brazilian population that customarily consume them together, and rice being the most frequently consumed food and highest contributor to energy intake. Results were presented as odds ratios (OR) and 95% confidence intervals (95% CI) for 10 g of each food grouping, considering a significance level of 5%.

## 3. Results

### 3.1. Survey Participant Characteristics

The characteristics of the population evaluated in ISA-Nutrition 2003, 2008, and 2015 are described in Table 1, according to body weight status. Over the three survey periods, the percentage of participants in the total population in the following categories increased across the three survey periods (2003, 2008, and 2015): older adults and adolescents, and participants who self-identified as non-white; with higher householder education; lower income; and who never drink alcohol or smoke. The prevalence of EBW in the period increased mostly among adolescents and adults. Besides the difference in the prevalence of EBW according to age group, in 2003, those self-identified as white, with higher income, and current smokers presented more EBW. In 2008, the higher EBW prevalence remained among those with higher income, but also in those who met the physical activity recommendations, and former smokers. In 2015, former smokers and those who consumed alcoholic beverages three or less times per week had more EBW. 

Regarding the dietary intake of the studied population, the total daily energy intake slightly increased while the total grams decreased, resulting in a marginally smaller dietary energy density from 2003 (1.26 kcal/g) to 2015 (1.22 kcal/g), especially in the ISA-Nutrition -2008 (1.19 kcal/g). Despite the decrease in energy density for total population, it has increased over the years for those with EBW. In the period, small magnitudes in the differences of the energy contribution were observed for protein (from 17.1% in ISA-Nutrition -2003 to 17.2% in ISA-Nutrition -2015) and carbohydrates (from 47.7% in ISA-Nutrition-2003 to 48.1% in ISA-Nutrition -2015). The Energy intake/Estimated Energy Requirement (EI/EER) ratio was higher for those without EBW, indicating less underreporting in this population.

### 3.2. Food Groupings Portion Sizes and Frequency of Intake

For total population, the portion sizes of white meat, salted snacks, coffee and tea, and eggs increased in the analyzed period and the portion sizes of rice, read meat, sweets, pasta, sandwiches, and cold cuts decreased (Table 2). Stratifying for age group (Tables S2–S4), there was a reduction in the portion size: of rice, fruits, commercial juices, tubers and roots, and cold cuts for adolescents; of flavored powder, sweets, sandwiches, and cold cuts for adults; and of fruits, pasta, cold cuts, and vegetables for older adults. In contrast, there was an increase in the portion size: of eggs among adolescents; of white meat, salted snacks, coffee and tea, and eggs among adults; and butter and margarine among older adults. The most consumed food grouping in the population was rice. The next foods ranked by percentage of consumers (>50%) varied according to the year of survey: in 2003, coffee and tea, breads and rolls, beans, milk, sugar, and red meat were the most consumed food groupings; in 2008, breads and rolls were more consumed than coffee and tea, and sugar and red meat were more consumed than milk; in 2015, compared to 2008, milk returned to being more consumed, followed by red meat, and sugar (Table 2). In the study period, there was an increase in the number of participants who reported the intake of rice, white meat, sweets, fruits, commercial juices, and toasts and biscuits (Table 2). For adolescents, there was higher frequency of intake of rice, red meat, sweets, fruits, flavored snacks, commercial juices, and toasts and biscuits, and a lower frequency of intake of sugar, milk, fruit juices, coffee and tea, and salted snacks. For adults, we observed a higher frequency of sweets, fruits, and commercial juices. For older adults, the frequency of alcoholic beverages, white meat, sweets, fruits, breads and rolls, commercial juices and legumes increased (Tables S2–S4).

### 3.3. Food Groupings Contribution to Total Energy Intake

The contribution to total energy intake of food groupings that contributed up to 90% of total amount of energy is described in Figure 1. The top three food groupings contributing to energy intake in all the years were rice, breads and rolls, and red meat; only for adolescents, red meat contributed more to energy than breads. The contribution of the other food groupings varied widely across the age groups. For example, in 2015, for adolescents, adults, and older adults respectively, toasts and biscuits were the 4th, 9th, and 11st; salted snacks were the 8th, 10th, and 18th; soft drinks were the 9th, 12th, and 19th; and milk and dairy were the 10th, 8th, and 6th in the ranking of energy contribution. 

Over the years, the energy contribution of rice and red meat decreased for total population, and the contribution of white meat, toasts and biscuits, sweets, fruits, and commercial juices increased. Significant changes occurred in the ranking of contribution from 2003 to 2015, such as white meat, which passed from 6th (4.3% of daily energy contribution) to the 4th place (5.5%) in the ranking, soft drinks, which ranked 12th in 2003 (3.2%), 5th in 2008 (4.4%), and 13th in 2015 (3.1%), and sweets, which passed from the 11th position in 2003 (3.5%) to the 7th in 2015 (4.4%). For adolescents, there was an increase in the contribution of toasts and biscuits, sweets, white meat, and commercial juices in the period, and a peak in soft drinks intake in 2008, followed by a reduction to the 2003 level in 2015. For adults, rice and breads had an important reduction in energy contribution, while pizza, salted snacks, and juices presented a reduction in 2008 but increased again in 2015. Butter and margarine, toasts and biscuits, and fruits constantly increased in the period. For older adults, there was a reduction in the energy contribution of rice and red meat, and an increase in white meat, sweets, butter and margarine, and alcoholic beverages. Moreover, the increase that occurred in 2008 for fruits returned to the same values of 2003 in 2015.

### 3.4. Association with Excess Body Weight

Table 3 describes the results of the logistic regression models evaluating the association between EBW and food portion size. Larger portion sizes of beans, cold cuts, fried snacks, fruit and commercial juices, pizza, red meat, rice, salted snacks, soft drinks, soups, and sugar were associated with higher odds of having EBW.

## 4. Discussion

Over the twelve years of study, there was diverse variation in food portion size intake in the population of São Paulo, with increases in some food groupings (white meat, salted snacks, coffee and tea, and eggs) and decreases in others (rice, red meat, sweets, pasta, sandwiches, and cold cuts). The percentage of people reporting the intake of six food groupings (rice, white meat, sweets, fruits, commercial juices, and toasts and biscuits) increased in the period. In this population, EBW was associated with larger portion sizes of 11 of the 30 food groupings investigated (cold cuts, fried snacks, fruit and commercial juices, pizza, red meat, rice, salted snacks, soft drinks, soups, and sugar).

Studies investigating trends in the food portion sizes at a population level in other countries also observed diversity in the patterns of increasing or decreasing portion sizes. In Ireland, significant increases were observed for ‘white sliced bread’, ‘brown/whole meal breads’, ‘all meat, cooked’, ‘poultry, roasted’ and ‘milk’, significant decreases were observed for ‘potatoes’, ‘chips/wedges’ and ‘ham, sliced’ and five foods did not change significantly over time ‘processed potato products’, ‘bacon/ham’, ‘cheese’, ‘yogurt’ and ‘butter/spreads’ [15]. In the USA, larger portion sizes were reported for several foods including soft drinks, coffee, tea, and ready-to-eat cereal, and smaller portion sizes were reported for fewer foods: margarine, mayonnaise, chicken, macaroni and cheese, and pizza [11]. Some of these results can be comparable to the findings from São Paulo, such as the increase of poultry in Ireland and coffee and tea in USA, and decrease of macaroni and cheese in USA similar to that of pasta in São Paulo. However, the differences illustrates that several aspects are related to the trends in portion sizes over time, such as culture, economy, social environment, food policies, among others. This highlights the importance of monitoring the trends in each specific population in order to support actions focused specifically for each group.

With respect to the energy contribution of the food groupings, there is a mixed pattern in the population: with an increase in contribution of food groupings related to healthier diet, such as fruits and white meat, in parallel to the reduction of red meat; but also increases of energy contribution of sweets, toasts and biscuits, and commercial juices. These data additionally reinforce the plurality of factors influencing food choices at the population level in terms of both portion size and eating frequency, such as access, taste, nutrition knowledge, cost, convenience, health perception, marketing, and social modelling [43,44,45], which result in changes over time occurring for both healthier and unhealthier foods. 

There is a pattern in the relationship between portion size and energy contribution of the food groupings. For some of them, such as fruits, commercial juices, and toasts and biscuits, there was an increase in the energy contribution, but not in portion size, and for sweets, the portion size decreased in parallel with an increase in energy contribution, indicating that these foods are likely more frequently consumed throughout the day. For other food groupings, such as white and red meat, rice, and pasta, the changes in portion sizes followed the changes in energy contribution, indicating less eating frequency in the same day. This observation is in accordance with previous studies investigating the relationship between EBW and eating occasions, specially snacking. They indicated that higher eating occasions is another important dietary factor linked to EBW, and must be more problematic than portion size alone. If a food is more frequently consumed, it must increase energy intake even if the portion size remains the same or decrease [46].

Despite many studies having investigated the relationship between food portion sizes and EBW, few have quantified this association in free-living populations. Two studies performed with Irish [16] and English [17] adults found a positive association between EBW and larger portions of “savory snacks, butter, full-fat spreads, meat products and dishes and chips and processed potatoes” and “whole and low-fat milks, potatoes, fresh meat, breads and rolls, low-fat spreads, vegetables, fish, chips and processed potatoes”, respectively. One study with British adolescents found that high-fiber breakfast cereal, cream, and high-energy soft drinks were positively associated with higher BMI after adjusting for misreporting, and larger portions of biscuits, cheese, cream, and cakes were associated with higher BMI after eliminating the effect of underreporting [18].

In a previous study, with 1005 adults from São Paulo in 2008, we observed a positive association between EBW and portion sizes of five food groupings: pizza, red meat, rice, salted snacks, and soft drinks [20]. In the present study, with larger sample size (*n* = 5270) and broader age range (12 years and older), we confirmed the previous associations, and observed additional food groupings: cold cuts, fried snacks, fruit and commercial juices, soups, and sugar. This fact indicates that a variety of food groupings may contribute to EBW. The larger sample size allowed for adjustment of additional confounding factors: in the first study, the portion size models were adjusted for dietary energy density, gender, age, *per capita* family income, physical activity level, and misreporting. In the present study, the models were further adjusted for racial self-identification, householder education, smoking status, alcoholic beverages intake, day of the week, intention to lose weight, number of eating occasions, and survey year. Although it is extremely complex to establish a direct link between portion size and EBW in free-living populations, given that many factors besides food portion size influences energy intake, such as number of eating occasions and energy density [47], adjusting for such factors allowed a more complex investigation of the association.

A cohort study with 120,877 U.S. women and men demonstrated that specific dietary factors are associated with long-term weight gain [48]. Increased daily servings of soft drinks, potatoes, red meat, refined grains (including rice), sweets and fruit juices were associated with greater weight gain. In the present study, similar associations were observed for the portion sizes, except for potatoes, which were classified in different ways in both studies, and for sweets, which were not statistically significant in the present study.

Food groupings with high energy and low nutrient density have consistently been associated with EBW [49]. In the present study, larger portions of fried snacks, salted snacks, and pizza are in accordance with this association. An issue of concern is that the portion size of salted snacks increased in the period in the total population and among adults, and the portion sizes of fried snacks and pizza did not decrease. In contrast, the number of adolescents reporting the intake of salted snacks decreased (from 26.3% in 2003 to 20.1% in 2015), although the prevalence of consumers is higher than in the other age groups.

In the present study, the portion size of rice, a traditional staple food in Brazil, was associated with EBW. Despite the consumption of rice concomitantly with beans has been consistently associated with healthier eating patterns [50,51], favorable weight outcomes in an 18-month randomized trial [52], and reduced BMI and waist circumference in low-income Brazilian women [53], studies indicate that the benefits of this pattern occur mainly because the intake of beans [42,54]. In accordance with our findings, other studies found a positive association between excess body weight and rice intake [42,55], but not with beans after adjusting for the highly-correlated and sizable rice intake [42], as we observed here. A study with 675 consumers in a restaurant serving buffet-by-weight also observed association between large portion size and EBW, even in consumers of rice and beans [56].

Beverage intake, particularly sugar-sweetened beverages, has also been related to EBW in previous studies [48,57,58], besides being an important risk factor for diabetes and triglycerides increase [59,60]. In the present study, the portion sizes of the analyzed beverages were associated with EBW (Fruit juices: OR = 1.014, *p* = 0.044; Commercial juices: OR = 1.020, *p* = 0.002; Soft drinks: OR = 1.011, *p* = 0.005), except coffee and tea (OR = 1.010, *p* = 0.059). Although the beverages food grouping did not discriminate if they were sugar-sweetened or not, the intake of sweeteners other than sugar is extremely low in this population: 90% of the population in 2008 consumed sugar-sweetened beverages. In addition, the portion size of sugar, frequently added to beverages, was also associated with EBW in the present study. Whereas moderate consumption of fruit juice may be an important source of vitamins, minerals, and antioxidants, excessive fruit juice consumption has been associated with weight gain [61], in accordance with the findings of the present study. Another issue regarding beverages in the present study is that regardless of the decrease in the portion size of commercial juices among adolescents over the survey years, the number of participants reporting the intake of this food grouping significantly increased, not only for adolescents, but also for adults and total population.

Similar to our findings, a study with Brazilian adults from the National Dietary Survey (NDS, *n* = 24,527) observed that the portion size of soft drinks was positively associated with EBW [21], as well as the study with adults from São Paulo in 2008 [20]. The three studies observed that the most frequently consumed beverage group by the population was coffee or tea (71.8% in ISA-Nutrition-2015 and 89.9% in NDS), which presented the smallest portion size among the beverages. There was less consumers of soft drinks in ISA-Nutrition-2015 (30.9%) than in NDS (35.4%), but more consumers of milk (59.2% vs. 31.3%), and alcoholic beverages (10.9% vs. 8.3%). In both studies, alcoholic beverages had the lowest frequency of consumption among the beverages, however they presented the largest portion size. The NDS study found positive association between EBW and portion size of alcoholic beverages (PR = 1.20, 95% CI, 1.11–1.29), different from the present study (OR = 1.003, 95% CI, 0.99–1.01). These discrepancies may result from methodological aspects of each research study or from characteristics of the population. Indeed, findings have been inconsistent in prior studies of alcohol intake and weight gain [48,62,63,64], and additional analyses are necessary to better understand this relationship.

Two other food groupings, red meats and cold cuts (considered processed meats), previously associated with EBW [48,65] and other diseases, such as some types of cancer [66], also had the portion size associated with EBW. These foods have been associated with worse diet quality, and have been considered an important source of excessive saturated and *trans* fats in the Brazilian diet [67]. 

Some limitations should be considered in the present study. First, the estimation of food portion size has fragilities, since it is based on self-report and is subject to recall bias, inherent to the dietary assessment method. However, a more accurate method, such as weighing, is impracticable in studies with a very large number of individuals, like the ISA-Nutrition. To minimize this error, we used tools to improve the dietary assessment quality, such as the use of figures as references for reporting the amounts of foods, the Multiple Pass Method, interviewer´s training, among others. Furthermore, we checked the standardization of the amounts for the household measurements to be sure that they were the same over the surveys. Secondly, the self-report of height and weight might have attenuated the association between BMI and food portion size if heavy individuals inform lower weights as compare to slim ones, however the self-reported data were validated in previous study [68], which observed high intraclass correlation between self-reported and measured parameters for weight (*r* > 0,94) and BMI (*r* > 0,85). Agreement between measured and self-report weight, height and BMI was good, as sensitivity was >91% and specificity was >83%. A third limitation is the systematic correction of the underreporting. Because underreporting is higher in people with higher BMI [69], it is one of the main problems when comparing dietary intake and EBW. It is strongly recommended that underreporting is taken into account in these associations to better interpret the data and results provided [40]. For this reason, we included in the models the Energy Intake/Estimated Energy Requirement ratio, a specific individual approach to statistically adjust for energy intake misreporting, instead of using cutoff methods, which could artificially elevate the association [40]. However, this method considers that all foods are equally underreported, despite that underreporting is frequently specific for certain foods, both in regard to consumed amount and to fidelity in reporting or not reporting consumption, according to the perception of being “unhealthy” or associated to obesity [70,71]. However, up to this moment, there is no methodology that considers this differentiation. The correlation between foods consumed together is a fourth limitation. The evaluation of the portion size of foods consumed together may attenuate the association of each food evaluated in a different regression model. Except for the model with the portion size of beans, in which portion size of rice was included due to its extremely high correlation of these two foods in this population, the analysis could not be performed with several food groupings at the same time, since only consumers presented a portion size value, and participants should have consumed all the foods included in the model to enable the analysis. 

Despite of the limitations, the present study represents the largest investigation of the relationship between EBW and food portion size, with three time points and a sampling design that represents all the population aged 12 years and older living in households in the urban area of the biggest city in Brazil and one of the most populous cities in the world, with more than 12 million habitants [72]. In addition, important confounders of this association were taken into account, such as energy density and number of eating occasions.

## 5. Conclusions

The present study identified lack of consistency in trends of food portion sizes and energy contribution over the twelve years of study in the population of São Paulo, with an increase in some food groupings in parallel with decrease in others. Portion sizes of 11 different food groupings were associated with EBW. Future interventions and public policies for optimal food intake in Brazil could be supported in light of these findings.

## Figures and Tables

**Table 1:** Characteristics of the population in the ISA-Nutrition in 2003, 2008 and 2015, according to body weight status ^a^.

	**ISA-Nutrition 2003**							**ISA-Nutrition 2008**						
	**Total Population** **(*n* = 2060)**		**Without EBW** **(*n* = 1427) ^b^**		**With EBW** **(*n* = 633) ^b^**			**Total Population** **(*n* = 1556)**		**Without EBW** **(*n* = 997) ^b^**		**With EBW** **(*n* = 559) ^b^**		
	**%**	**95% CL**	**%**	**95% CL**	**%**	**95% CL**	***p*^c^**	**%**	**95% CL**	**%**	**95% CL**	**%**	**95% CL**	***p*^c^**
**Age group**														
**Adolescents (12–19 years)**	17.5	(15.6, 19.6)	82.2	(82.8, 89.1)	13.8	(10.9, 17.2)		24.3	(21.2, 27.6)	75.2	(70.7, 79.3)	24.8	(20.7, 29.3)	
**Adults (20–59 years)**	70.1	(68.0, 72.1)	59.0	(55.5, 62.6)	41.0	(37.4, 44.6)		62.6	(58.9, 66.1)	53.1	(48.7, 57.4)	46.9	(42.6, 51.3)	
**Older adults (60 years or more)**	12.4	(10.8, 14.2)	64.1	(59.2, 68.6)	36.0	(31.4, 40.8)	0.000	13.2	(11.0, 15.7)	66.0	(61.9, 69.8)	34.0	(30.2, 38.1)	0.000
**Gender**														
**Female**	52.4	(49.5, 55.2)	66.5	(62.8, 69.9)	33.6	(30.1, 37.2)		54.3	(51.8,.56.8)	61.8	(57.8, 65.5)	38.3	(34.5, 42.2)	
**Male**	47.6	(44.8, 50.4)	62.1	(57.7, 66.3)	37.9	(33.7, 42.3)	0.145	45.7	(43.1, 48.2)	58.2	(53.3, 63.0)	41.8	(37.0, 46.7)	0.259
**Racial Self-identification**														
**White**	67.2	(63.2,71.0)	61.9	(58.7, 65.1)	38.1	(34.9, 41.3)		60.5	(54.7, 65.9)	59.5	(55.7, 63.2)	40.5	(36.8, 44.3)	
**Non white**	32.8	(29.1, 36.8)	69.6	(65.4, 73.5)	30.4	(26.5, 34.6)	0.004	39.5	(34.0, 45.3)	61.2	(55.4, 66.7)	38.8	(33.3, 44.6)	0.435
**Education of parent/householder**														
**≤9 years**	54.0	(49.5, 58.4)	65.3	(61.8, 68.7)	34.7	(31.3, 38.2)		46.8	(40.4, 53.2)	60.7	(56.1, 65.1)	39.3	(34.9, 43.9)	
**>9 years**	46.0	(41.6, 50.5)	63.5	(58.9, 67.8)	36.5	(32.2, 41.1)	0.521	53.3	(46.8, 59.6)	59.5	(55.5, 63.4)	40.5	(36.6, 44.5)	0.675
**Household income ^d^**														
**≤1 MW ^d^**	38.5	(34.0, 43.2)	68.4	(63.6, 72.9)	31.6	(27.1, 36.4)		44.3	(39.1, 49.5)	65.6	(61.3, 69.7)	34.4	(30.3, 38.7)	
**>1 MW ^d^**	61.5	(56.8, 66.0)	61.9	(58.4, 65.3)	38.1	(34.7, 41.6)	0.041	55.7	(50.4, 60.9)	55.8	(51.9, 59.6)	44.2	(40.4, 48.1)	0.000
**Leisure-time Physical Activity Level**														
**Meet the recommendation**	22.7	(19.9, 25.8)	62.2	(56.8, 67.3)	37.8	(32.7, 43.2)		22.2	(20.3, 24.4)	57.4	(50.3, 64.2)	42.6	(35.8, 50.7)	
**Don’t meet the recommendation**	77.3	(74.2, 80.1)	65.0	(62.0, 67.9)	35.0	(32.1, 38.0)	0.339	77.8	(75.6, 79.7)	61.1	(57.5, 64.5)	38.9	(35.5, 42.5)	0.362
**Global Physical Activity Level**														
**Meet the recommendation**	77.2	(74.0, 80.1)	65.8	(61.1, 70.3)	34.2	(29.7, 38.9)		79.8	(77.0, 82.4)	58.4	(55.1, 61.7)	41.6	(38.3, 44.9)	
**Don’t meet the recommendation**	22.8	(19.9, 26.0)	64.1	(61.0, 67.1)	35.9	(32.9, 39.0)	0.517	20.2	(17.6, 23.0)	67.0	(61.0, 72.4)	33.0	(27.6, 39.0)	0.008
**Smoking status**														
**Never**	65.9	(62.9, 68.8)	65.8	(62.4, 69.0)	34.2	(31.0, 37.6)		66.4	(63.1, 69.5)	63.2	(59.7, 66.5)	36.8	(33.5, 40.3)	
**Former**	19.2	(16.6, 22.0)	70.4	(63.1, 76.8)	29.6	(23.2, 36.9)		15.4	(12.9, 18.3)	46.7	(39.0, 54.5)	53.3	(45.5, 61.0)	
**Current**	15.0	(12.4, 17.9)	50.1	(52.4, 57.3)	49.9	(42.6, 57.2)	0.000	18.2	(15.4, 21.4)	60.5	(53.4, 67.1)	39.5	(32.9, 46.6)	0.000
**Alcohol intake**														
**Never**	46.1	(42.5, 49.7)	67.4	(63.4, 71.2)	32.6	(28.8, 36.6)		50.0	(46.7, 53.2)	63.4	(59.3, 67.3)	36.6	(32.7, 40.7)	
**≤3 times per week**	48.6	(45.0, 52.2)	62.0	(57.9, 65.9)	38.0	(34.1, 42.1)		44.8	(41.6, 48.1)	57.3	(52.2, 62.2)	42.7	(37.8, 47.8)	
**>3 times per week**	5.32	(4.10, 6.88)	57.6	(44.1, 70.1)	42.4	(29.9, 55.9)	0.110	5.23	(4.0, 6.8)	54.7	(42.5, 66.4)	45.3	(33.6, 57.5)	0.094
	**mean**	**IQR**	**mean**	**IQR**	**mean**	**IQR**	***p*^c^**	**mean**	**IQR**	**mean**	**IQR**	**mean**	**IQR**	***p*^c^**
**Dietary intake**														
**Total energy (kcal/day)**	1826	(1235, 2304)	1849	(1269, 2290)	1786	(1204, 2310)	0.214	1932	(1256, 2430)	1994	(1318, 2488)	1838	(1177, 2321)	0.002
**EI/EER**	0.71	(0.49, 0.87)	0.74	(0.50, 0.92)	0.65	(0.46, 0.81)	0.000	0.77	(0.50, 0.96)	0.82	(0.55, 1.02)	0.69	(0.44, 0.84)	0.000
**Total grams (g/day)**	1547	(1041, 1847)	1517	(1065, 1833)	1602	(998, 1900)	0.461	1666	(1130, 2047)	1699	(1161, 2067)	1616	(1075, 1999)	0.037
**Total energy density (kcal/g)**	1.26	(1.00, 1.47)	1.26	(1.00, 1.47)	1.25	(0.98, 1.47)	0.479	1.19	(0.97, 1.36)	1.21	(1.00, 1.38)	1.17	(0.95, 1.35)	0.026
**Protein (%kcal)**	17.1	(13.3, 19.9)	16.9	(13.1, 19.8)	17.5	(13.6, 20.4)	0.102	17.4	(13.2, 10.4)	17.1	(13.2, 19.8)	17.7	(13.2, 21.0)	0.074
**Carbohydrates (%kcal)**	47.7	(41.48, 53.7)	48.4	(42.5, 53.9)	46.3	(40.2, 52.9)	0.002	47.5	(41.5, 54.3)	47.8	(41.6, 54.5)	47.0	(40.6, 54.2)	0.425
**Total Fat (%kcal)**	32.7	(27.3, 38.3)	32.4	(26.9, 37.8)	33.2	(28.0, 38.9)	0.097	32.4	(26.5, 38.0)	32.4	(26.7, 38.0)	32.3	(26.3, 38.2)	0.910
	**ISA-Nutrition 2015**							***p*-Values for the Difference Across Surveys ^c^**						
	**Total Population** **(*n* = 1654)**		**Without EBW** **(*n* = 969) ^b^**		**With EBW** **(*n* = 685) ^b^**									
	**%**	**95% CL**	**%**	**95% CL**	**%**	**95% CL**	***p*^c^**	**Total Population**		**Without EBW**		**With EBW**		
**Age group**														
**Adolescents (12–19 years)**	22.8	(20.5, 25.3)	70.6	(65.9, 74.9)	29.4	(25.2, 34.1)								
**Adults (20–59 years)**	54.3	(51.5, 57.0)	45.8	(41.5, 50.2)	54.2	(49.8, 58.5)								
**Older adults (60 years or more)**	22.9	(20.1, 26.0)	63.1	(58.3, 67.6)	36.9	(32.4, 41.7)	0.000	0.000		0.000		0.000		
**Gender**														
**Female**	49.7	(46.8, 52.7)	54.5	(50.9, 58.1)	45.5	(41.9, 49.1)								
**Male**	50.3	(47.3, 53.2)	56.3	(52.1, 60.4)	43.7	(39.6, 47.9)	0.482	0.112		0.054		0.673		
**Racial Self-identification**														
**White**	50.4	(46.5, 54.2)	53.5	(49.1, 57.9)	46.5	(42.1, 50.9)								
**Non white**	49.7	(45.8, 53.5)	57.2	(53.1, 61.1)	42.8	(38.9, 46.9)	0.233	0.000		0.000		0.000		
**Education of parent/householder**														
**≤9 years**	45.0	(40.8, 49.3)	56.2	(52.4, 59.9)	43.8	(40.1, 47.6)								
**>9 years**	55.0	(50.7, 59.2)	54.4	(49.6, 59.2)	45.6	(40.8, 50.4)	0.586	0.028		0.056		0.099		
**Household income ^d^**														
**≤1 MW ^d^**	49.9	(44.8, 54.9)	54.3	(50.2, 58.2)	45.8	(41.8, 49.8)								
**>1 MW ^d^**	50.1	(45.1, 55.2)	53.8	(48.7, 58.7)	46.3	(41.3, 51.3)	0.881	0.007		0.031		0.003		
**Leisure-time Physical Activity Level**														
**Meet the recommendation**	21.9	(19.5, 24.6)	58.2	(51.7, 64.5)	41.8	(35.5, 48.3)								
**Don’t meet the recommendation**	78.1	(75.4, 80.5)	54.6	(51.7, 57.6)	45.4	(42.4, 48.3)	0.286	0.883		0.755		0.407		
**Global Physical Activity Level**														
**Meet the recommendation**	74.6	(71.6, 77.4)	59.4	(53.8, 64.7)	40.6	(35.3, 46.2)								
**Don’t meet the recommendation**	25.4	(22.6, 28.4)	54.3	(50.9, 57.6)	45.7	(42.4, 49.1)	0.130	0.076		0.209		0.050		
**Smoking status**														
**Never**	71.0	(68.1, 73.7)	56.4	(53.0, 59.8)	43.6	(40.2, 47.0)								
**Former**	15.0	(12.8, 17.6)	44.3	(37.8, 50.9)	55.7	(49.1, 62.2)								
**Current**	14.0	(12.1, 16.1)	62.0	(54.1, 69.4)	38.0	(30.6, 45.9)	0.002	0.023		0.000		0.013		
**Alcohol intake**														
**Never**	58.6	(54.5, 62.6)	58.8	(55.2, 62.3)	41.2	(37.7, 44.8)								
**≤3 times per week**	38.9	(35.0, 42.8)	50.1	(44.8, 55.3)	49.9	(44.7, 55.2)								
**>3 times per week**	2.55	(1.70, 3.79)	61.1	(45.3, 74.9)	38.9	(25.1, 54.7)	0.010	0.000		0.000		0.003		
	**mean**	**IQR**	**mean**	**IQR**	**mean**	**IQR**	***p*^c^**	**Total Population**		**Without EBW**		**With EBW**		
***Dietary intake***														
**Total energy (kcal/day)**	1877	(1262, 2344)	1952	(1285, 2472)	1783	(1226, 2208)	0.002	0.00		0.01		0.84		
**EI/EER**	0.72	(0.49, 0.89)	0.78	(0.54, 0.96)	0.65	(0.45, 0.78)	0.000	0.00		0.00		0.97		
**Total grams (g/day)**	1615	(1110, 1967)	1640	(1139, 2018)	1585	(1082, 1913)	0.021	0.02		0.00		0.15		
**Total energy density (kcal/g)**	1.22	(0.98, 1.40)	1.24	(1.00, 1.43)	1.19	(0.97, 1.36)	0.040	0.01		0.08		0.03		
**Protein (%kcal)**	17.2	(13.2, 20.0)	17.2	(13.1, 20.0)	17.1	(13.2, 20.0)	0.767	0.04		0.51		0.34		
**Carbohydrates (%kcal)**	48.1	(42.3, 54.1)	48.5	(42.4, 55.2)	47.6	(42.2, 52.8)	0.033	0.00		0.68		0.08		
**Total Fat (%kcal)**	31.8	(26.7, 37.1)	31.5	(26.3, 36.7)	32.2	(27.3, 37.5)	0.115	0.11		0.05		0.03		

Abbreviations: EBW, Excess body weight (includes overweight and obesity); 95% CL, 95% Confidence Limits; MW, Minimum Wage; EI/EER, Energy intake/Estimated Energy Requirement ratio; IQR, Interquartile Range. ^a^ All the analyses took into account the survey sampling design. ^b^ Without EBW: BMI < p85; With EBW: BMI ≥ p85 for adolescents [30]. Without EBW: BMI < 25 kg/m^2^; With EW: BMI ≥ 25 kg/m^2^ for adults [31]. Without EBW: BMI < 28 kg/m^2^; With EBW: BMI ≥ 28 kg/m^2^ for older adults [32]. ^c^ Categorical variables were compared using Chi Square tests and continuous variables were compared using Mann-Whitney tests. ^d^ Values were adjusted by the number of individual in the household. One MW is approximately 78 US dollars in 2003, 217 US dollars in 2008, and 236 US dollars in 2015.

**Table 2:** Frequency of consumption and portion size of food groupings for total population of the ISA-Nutrition in 2003, 2008 and 2015.

**Total Population (*n* = 5270)**										
	**ISA-Nutrition 2003 (*n* = 2060)**					**ISA-Nutrition 2008 (*n* = 1556)**				
**Food Grouping ^a^**	**Consumption**		**Portion Size (g)**			**Consumption**		**Portion Size (g)**		
	**%**	**SE**	**Mean**	**Median**	**IQR**	**%**	**SE**	**Mean**	**Median**	**IQR**
Alcoholic beverages	9.3	0.01	732.8	542.4	(602.6, 862.9)	10.2	0.01	726.1	477.2	(590.0, 862.1)
Beans	63.3	0.02	104.3	86.0	(98.6, 110.1)	65.8	0.02	108.6	86.0	(103.2, 114.0)
Breads and rolls	71.4	0.01	62.0	50.0	(59.6, 64.4)	70.9	0.01	63.8	50.0	(61.1, 66.6)
Butter and margarine	47.1	0.02	16.2	15.0	(14.4, 18.0)	41.5	0.02	18.4	15.0	(16.7,20.0)
Cakes	10.4	0.01	91.5	60.2	(76.7, 106.2)	11.0	0.01	90.0	70.0	(79.5, 100.4)
Cheese	25.6	0.02	37.8	30.0	(34.3, 41.4)	25.6	0.02	37.8	30.0	(34.4, 41.2)
Coffee and tea	75.5	0.01	117.6	96.1	(112.5, 122.7)	69.3	0.02	116.8	97.6	(109.9, 123.8)
Cold Cuts	11.6	0.01	40.1	30.0	(34.2, 46.0)	16.2	0.02	33.9	30.0	(29.5, 38.4)
Commercial juices	17.0	0.01	312.3	257.1	(289.3, 335.2)	25.3	0.02	290.9	240.0	(274.9, 306.9)
Eggs	9.3	0.01	56.5	50.0	(52.8, 60.2)	10.5	0.01	49.4	50.0	(42.6, 56.2)
Flavored powder	12.1	0.01	30.3	25.0	(27.1, 33.6)	12.4	0.01	27.3	24.0	(23.6, 30.9)
Flavored snacks	2.5	0.00	75.4	50.0	(35.2, 115.5)	3.4	0.01	74.9	60.0	(56.0, 93.8)
Fried snacks	5.7	0.01	117.6	100.0	(101.3, 133.9)	6.0	0.01	145.8	100.0	(118.4, 173.3)
Fruit juices	20.8	0.01	446.9	251.6	(155.4, 738.5)	20.1	0.01	268.5	240.4	(248.9, 288.0)
Fruits	28.8	0.02	163.4	135.0	(152.6, 174.1)	34.1	0.02	172.4	142.0	(157.6, 187.3)
Leafy Vegetables	40.2	0.02	40.5	30.0	(36.4, 44.6)	40.3	0.01	41.6	30.0	(37.6, 45.7)
Milk	62.7	0.01	170.4	148.5	(161.8, 178.9)	58.5	0.02	162.8	128.9	(154.3, 171.3)
Pasta	19.9	0.01	238.5	238.8	(219.7, 257.2)	19.3	0.01	214.8	208.8	(198.0, 231.6)
Pizza	7.2	0.01	299.7	286.5	(253.1, 346.2)	7.7	0.01	229.3	190.3	(196.3, 262.3)
Red meat	57.1	0.01	112.5	100.0	(107.3, 117.6)	59.9	0.02	116.2	100.0	(107.3, 125.0)
Rice	78.3	0.02	160.8	132.5	(152.5,169.2)	80.8	0.02	145.0	124.0	(137.3, 152.6)
Salted snacks	17.7	0.01	97.5	71.3	(84.9, 110.1)	16.4	0.01	102.3	73.2	(86.6, 117.9)
Sandwiches	4.3	0.01	223.5	200.0	(196.0, 250.9)	4.9	0.01	169.5	158.9	(142.6, 196.4)
Soft drinks	32.6	0.01	340.4	302.8	(319.4, 361.3)	38.8	0.02	327.2	250.0	(308.8, 345.6)
Soups	9.9	0.02	414.6	329.4	(344.3, 484.9)	4.8	0.01	402.9	325.0	(331.1, 474.8)
Sugar	57.2	0.02	10.1	7.7	(9.28, 11.0)	63.9	0.02	10.2	8.0	(9.49, 10.8)
Sweets	20.7	0.01	87.7	66.7	(80.3, 95.1)	29.2	0.02	84.1	56.0	(75.0, 93.2)
Toast and Biscuits	24.1	0.01	47.1	32.0	(42.1, 52.0)	26.9	0.01	49.6	30.0	(43.8, 55.4)
Tubers and roots	13.9	0.01	118.7	90.0	(101.2, 136.2)	15.6	0.01	132.7	85.0	(92.5, 172.8)
Vegetables	43.1	0.02	75.7	60.0	(68.7, 82.8)	42.6	0.02	79.2	52.5	(67.4, 90.8)
White meat	33.0	0.02	99.3	80.0	(91.3, 107.3)	37.1	0.01	116.5	100.0	(104.1, 128.8)
**Total Population (*n* = 5270)**										
	**ISA-Nutrition 2015 (*n* = 1654)**					***p*-Values for Difference Across the Surveys ^b^**				
**Food Grouping ^a^**	**Consumption**		**Portion Size (g)**							
	**%**	**SE**	**Mean**	**Median**	**IQR**	**Frequency**	**Portion Size**			
Alcoholic beverages	10.9	0.01	681.8	351.5	(514.9, 848.7)	0.278	0.638			
Beans	66.9	0.02	107.3	86.0	(102.6, 112.1)	0.173	0.409			
Breads and rolls	75.2	0.01	58.9	50.0	(56.7, 61.1)	0.057	0.086			
Butter and margarine	47.9	0.01	17.4	15.0	(16.5, 18.3)	0.913	0.229			
Cakes	12.4	0.01	90.0	60.8	(76.3, 103.7)	0.175	0.881			
Cheese	26.6	0.01	35.7	30.0	(32.5, 38.8)	0.673	0.390			
Coffee and tea	71.8	0.01	143.8	125.1	(135.1, 152.5)	0.038	0.000			
Cold Cuts	12.6	0.01	28.7	20.0	(24.2, 33.2)	0.369	0.003			
Commercial juices	28.8	0.02	281.9	240.0	(261.8, 302.0)	0.000	0.054			
Eggs	11.9	0.01	70.1	50.0	(62.5, 77.7)	0.075	0.002			
Flavored powder	11.8	0.01	27.7	24.0	(24.1, 31.4)	0.854	0.262			
Flavored snacks	3.7	0.01	58.9	48.5	(47.6, 70.2)	0.066	0.415			
Fried snacks	5.0	0.01	106.7	100.0	(92.8, 120.7)	0.530	0.565			
Fruit juices	21.1	0.01	287.0	251.6	(266.8, 307.1)	0.904	0.277			
Fruits	45.5	0.02	156.0	131.0	(147.0, 164.9)	0.000	0.258			
Leafy Vegetables	42.3	0.02	37.2	30.0	(34.3, 40.1)	0.445	0.224			
Milk	59.2	0.02	164.9	148.5	(157.9, 171.9)	0.107	0.297			
Pasta	19.2	0.01	192.1	168.2	(168.5, 215.7)	0.718	0.003			
Pizza	6.7	0.01	285.9	302.1	(249.4, 322.4)	0.740	0.507			
Red meat	58.7	0.02	103.2	92.5	(97.3, 109.0)	0.413	0.027			
Rice	82.6	0.01	146.0	125.0	(138.7, 153.3)	0.052	0.007			
Salted snacks	15.8	0.01	119.6	100.0	(106.5, 132.6)	0.264	0.022			
Sandwiches	3.3	0.01	170.4	158.9	(142.4, 198.3)	0.300	0.007			
Soft drinks	30.9	0.01	326.3	275.1	(307.2, 345.3)	0.594	0.312			
Soups	7.4	0.01	388.4	392.1	(338.2, 438.6)	0.183	0.561			
Sugar	55.1	0.02	10.9	8.1	(9.93, 11.8)	0.614	0.279			
Sweets	34.2	0.02	70.5	45.0	(62.6, 78.3)	0.000	0.002			
Toast and Biscuits	28.0	0.01	53.8	30.0	(47.1, 60.5)	0.040	0.112			
Tubers and roots	16.2	0.01	131.1	80.0	(107.4, 154.7)	0.153	0.396			
Vegetables	46.9	0.02	71.4	52.0	(65.3, 77.6)	0.11	0.399			
White meat	37.7	0.01	111.8	100.0	(104.5, 119.1)	0.031	0.021			

Abbreviations: SE, Standard Error; IQR, Interquartile Range. ^a^ The list of foods included in each food grouping is described in Table S1. ^b^ Trend was estimated using logistic and linear regression models.

**Table 3:** Association of food groupings’ portion size for the total population in the Health Survey of São Paulo with excess body weight ^a^ assessed using logistic regression model.

Food Grouping ^b^		Adjusted Model for Portion Size ^c^		
	*n* ^d^	OR	95% CI	*p*
Alcoholic beverages	381	1.003	(0.99, 1.01)	0.276
Beans ^e^	3236	1.014	(1.00, 1.03)	0.148
Breads and rolls	3514	1.024	(0.99, 1.06)	0.155
Butter and margarine	2215	1.058	(0.96, 1.16)	0.239
Cakes	510	1.005	(0.97, 1.04)	0.774
Cheese	1161	1.046	(0.98, 1.11)	0.154
Coffee and tea	3469	1.010	(1.00, 1.02)	0.059
Cold cuts	581	1.112	(1.04, 1.19)	**0.004**
Commercial juices	1066	1.020	(1.01, 1.03)	**0.002**
Eggs	504	1.096	(1.00, 1.21)	0.062
Flavored powder	635	1.017	(0.94, 1.11)	0.699
Flavored Snacks	171	1.037	(1.00, 1.07)	0.278
Fried snacks	252	1.056	(1.00, 1.11)	**0.030**
Fruit juices	919	1.014	(1.00, 1.03)	**0.044**
Fruits	1795	1.004	(0.99, 1.02)	0.440
Leafy vegetables	1927	1.018	(0.98, 1.06)	0.377
Milk	3012	1.001	(0.99, 1.01)	0.867
Pasta	887	1.013	(1.00, 1.03)	0.081
Pizza	290	1.028	(1.01, 1.05)	**0.007**
Red meat	2779	1.017	(1.00, 1.03)	**0.027**
Rice	3951	1.025	(1.01, 1.04)	**0.000**
Salted snacks	799	1.031	(1.01, 1.06)	**0.010**
Sandwiches	172	0.989	(0.93, 1.05)	0.711
Soft drinks	1589	1.011	(1.00, 1.02)	**0.005**
Soups	376	1.016	(1.00, 1.03)	**0.010**
Sugar	2793	1.148	(1.02, 1.29)	**0.020**
Sweets	1260	1.009	(0.99, 1.03)	0.407
Toast and biscuits	1286	1.009	(0.98, 1.04)	0.578
Tubers and roots	704	1.011	(0.99, 1.03)	0.187
Vegetables	1997	1.016	(1.00, 1.03)	0.089
White meat	1624	0.999	(0.98, 1.02)	0.882

OR, estimated regression coefficient for 10 grams of each food grouping; 95% CI, 95% Confidence Interval. ^a^ Without EBW: BMI < p85; With EBW: BMI ≥ p85 for adolescents [30]. Without EBW: BMI <25 kg/m^2^; With EBW: BMI ≥ 25 kg/m^2^ for adults [31]. Without EBW: BMI < 28 kg/m^2^; With EBW: BMI ≥ 28 kg/m^2^ for older adults [32]. ^b^ The list of foods included in each food grouping is described in Table S1. ^c^ Models adjusted for dietary energy density (kcal/g), age (years), gender (male or female), racial self-identification (white or non-white), household income (≤1 or >1 minimal wage per person in the family), householder education (≤9 or >9 years), smoking status (never, former, or current smoker), alcoholic beverages intake (never, ≤3 times per week, or >3 times per week), physical activity level (meet or do not meet WHO recommendation), Energy intake/Estimated Energy Requirement ratio, day of the week (weekday or weekend), intention to lose weight (yes or no), number of eating occasions, and survey year (2003, 2008, or 2015). ^d^ Sample size for each regression model. ^e^ Models for beans portion sizes were further adjusted by rice portion sizes, due to the high correlation between these variables. In bold, *p*-values < 0.05.

## Supplementary Materials

The following are available online at http://www.mdpi.com/2072-6643/10/6/696/s1, Table S1: List of foods that were included in each food grouping; Table S2: Frequency of consumption and portion size of food groupings for adolescents of the Health Survey of São Paulo in 2003, 2008 and 2015; Table S3: Frequency of consumption and portion size of food groupings for adults of the Health Survey of São Paulo in 2003, 2008 and 2015; Table S4: Frequency of consumption and portion size of food groupings for older adults of the Health Survey of São Paulo in 2003, 2008 and 2015.
