# Supplementary material for: Differences over 12 Years in Food Portion Size and Association with Excess Body Weight in the City of São Paulo, Brazil
Source: Nutrients. 2018 May 30;10(6):696. doi: 10.3390/nu10060696 (PMC6024366; doi:10.3390/nu10060696)
Supplement: Supplementary file 1 [file nutrients-10-00696-s001.pdf]

Table S1. List of foods that were included in each food grouping.

Food groupings	Description of food group contents
Alcoholic beverages	Fermented and distilled alcoholic beverages
Beans	Black and brown beans
Breads and rolls	White and brown bread and rolls
Butter and margarine	Butter and margarine
Cakes	Cakes with or without topping and filling
Cheese	Cheeses, all types
Coffe and tea	Coffee and tea, caffeinated or decaffeinated
Cold Cuts	Ham, salami, roast beef, turkey ham
Commercial juices	Ready-to-drink juices, powder juices, nectar, etc. With or without sugar
Eggs	Boiled, fries, scrambled eggs
Flavored powder	Artificially flavored powders: chocolate, strawberry, etc.
Flavored Snacks	Potato chips, flavored snacks
Fried Snacks	French fries, onion rings, fried cornmeal mush
Fruits	Banana, apple, papaya, melon, mango, citrus fruit, etc.
Fruit juices	Lemonade, pineapple, orange, passion fruit juices, etc. With or without sugar
Leafy vegetables	Lettuce, cabbage, escarole, kale, etc.
Milk	Whole, lowfat, skimmed and fortified milks
Pasta	Spaghetti, ravioli, noodels, canelloni, lasagna, etc. With or without sauce
Pizza	Sweet (e.g., chocolate) or salt (e.g., mozzarella) pizza
Red meat	Beef, hamburger, liver, ribs, jerked beef, lamb, pork, etc.
Rice	White and brown rice
Salted snacks	Fried or baked savory snacks, e.g., "esfiha", "coxinha", croissant, cheese bread, etc.
Sandwiches	Hamburguer, cheeseburguer, cold cut sandwiches, etc.
Soft drinks	Fruit-flavored drinks, cola and noncola soft drinks, light and diet soft drinks
Soups	Soups and creams, including with vegetables or pasta
Sugar	Added sugar, white or brown
Sweets	Chocolate, puddings, ice creams, sweet rolls and pies, etc.
Toast and biscuits	White and brown toasts, savory or sweet biscuits and crackers, with or without filling
Tubers and roots	Potato, cassava, yams, sweet potato
Vegetables	Carrots, tomato, eggplant, broccoli, cucumber, radish, etc.
White meat	Chicken, turkey, fish and seafood

Table S2. Frequency of consumption and portion size of food groupings for adolescents of the ISA-Nutrition in 2003, 2008 and 2015.

	Total Adolescents (*n* = 1711)									
	ISA-Nutrition 2003 (*n* = 677)					ISA-Nutrition 2008 (*n* = 512)				
**Food Grouping** [a]	**Consumption**		**Portion size (g)**			**Consumption**		**Portion size (g)**		
	**%**	**SE**	**mean**	**median**	**IQR**	**%**	**SE**	**mean**	**median**	**IQR**
Alcoholic beverages	3.3	0.01	864.4	582.9	.	4.3	0.01	985.2	703.0	.
Beans	65.1	0.03	118.2	86.0	(112.2, 124.3)	63.6	0.03	120.0	86.0	(113.5, 126.6)
Breads and rolls	74.8	0.02	71.4	50.0	(67.9, 75.0)	68.2	0.02	76.1	60.0	(71.4, 80.7)
Butter and margarine	50.3	0.03	22.1	15.0	(18.6, 25.6)	40.4	0.02	24.1	22.5	(21.9, 26.3)
Cakes	13.2	0.02	94.1	72.0	(82.5, 105.7)	14.3	0.02	104.2	90.0	(79.9, 128.5)
Cheese	23.6	0.02	34.7	30.0	(30.6, 38.9)	21.9	0.02	39.5	27.0	(33.4, 45.6)
Coffee and tea	49.8	0.03	125.5	120.1	(112.9, 138.2)	42.3	0.03	130.8	120.1	(119.3, 142.3)
Cold Cuts	14.6	0.01	37.2	30.0	(29.3, 45.2)	18.4	0.02	36.5	30.0	(30.8, 42.1)
Commercial juices	22.1	0.02	330.1	308.6	(303.9, 356.3)	35.2	0.02	322.4	252.6	(297.1, 347.7)
Eggs	11.1	0.01	55.8	50.0	(48.2, 63.3)	6.8	0.01	59.9	50.0	(50.1, 69.6)
Flavored powder	26.9	0.02	28.2	25.0	(24.8, 31.5)	25.8	0.02	27.6	27.5	(24.9, 30.4)
Flavored Snacks	5.0	0.01	68.9	55.0	(47.9, 89.9)	9.0	0.01	85.9	66.0	(70.3,101.6)
Fried snacks	7.3	0.01	111.6	100.0	(91.4, 131.7)	8.8	0.01	150.0	106.4	(100.5, 199.6)
Fruit juices	24.0	0.02	298.1	251.6	(277.8, 318.3)	19.7	0.02	296.4	251.6	(270.3, 322.4)
Fruits	20.6	0.02	192.3	150.0	(163.7, 220.9)	19.7	0.02	149.9	135.0	(131.0, 168.8)
Leafy Vegetables	30.5	0.02	35.2	30.0	(31.6, 38.7)	29.2	0.02	36.2	30.0	(31.6, 40.8)
Milk	59.5	0.03	201.9	198.0	(188.3, 215.4)	54.3	0.02	213.3	198.0	(197.6, 228.9)
Pasta	22.3	0.02	237.8	225.0	(208.9, 266.7)	20.8	0.02	218.3	208.8	(195.6, 241.0)
Pizza	6.3	0.01	296.6	286.5	(228.1, 365.2)	9.5	0.02	282.2	200.3	(179.5, 385.0)
Red meat	54.5	0.03	104.8	100.0	(98.0, 111.5)	61.8	0.02	126.4	100.0	(108.8, 144.0)
Rice	78.5	0.02	169.1	150.0	(155.1, 183.0)	76.6	0.02	162.7	135.0	(153.9, 171.5)
Salted snacks	26.3	0.02	108.1	72.0	(89.8, 126.3)	22.9	0.02	126.6	94.0	(105.2, 148.0)
Sandwiches	6.5	0.01	199.4	200.0	.	8.9	0.01	186.1	186.0	.
Soft drinks	44.1	0.03	345.1	311.4	(320.5, 369.6)	57.6	0.03	367.9	307.2	(342.1, 393.8)
Soups	7.5	0.02	418.4	330.0	.	1.5	0.01	629.0	702.4	.
Sugar	53.4	0.02	12.7	10.0	(11.1, 14.3)	55.2	0.03	12.7	12.0	(11.6, 13.8)

Sweets	28.5	0.02	82.7	56.2	(69.6, 95.8)	37.0	0.03	91.7	61.1	(77.9, 105.6)
Toast and Biscuits	27.4	0.02	72.4	54.0	(64.3, 80.4)	31.2	0.02	82.4	52.7	(71.6, 93.2)
Tubers and roots	14.0	0.02	145.1	90.0	(103.2, 187.0)	11.9	0.01	128.5	90.0	(86.8, 170.3)
Vegetables	32.5	0.02	59.2	45.0	(51.8, 66.7)	26.9	0.02	68.2	50.0	(58.7, 77.7)
White meat	32.0	0.02	87.9	75.0	(75.7, 100.0)	30.2	0.02	110.7	90.0	(94.7, 126.7)

Abbreviations: SE, Standard Error; IQR, Interquartile Range. [a] The list of foods included in each food grouping is described in Table S1. [b] Trend was estimated using logistic and linear regression models. Note: missing standard errors because of stratum with single sampling unit.

Table S2. (Cont.) Frequency of consumption and portion size of food groupings for adolescents of the ISA-Nutrition in 2003, 2008 and 2015.

**Food Grouping [a]**	**Total Adolescents (n = 1711)**						
	**ISA-Nutrition 2015 (n = 522)**						
	**Consumption**		**Portion size (grams)**			**p [b]**	
	**%**	**SE**	**mean**	**median**	**IQR**	**trend portion**	**trend freq**
Alcoholic beverages	2.0	0.01	687.5	564.0	.	.	0.198
Beans	70.8	0.02	115.0	106.0	(107.7, 122.2)	0.487	0.139
Breads and rolls	70.2	0.02	66.8	50.0	(62.8, 70.8)	0.101	0.119
Butter and margarine	44.9	0.02	19.3	15.0	(16.8, 21.8)	0.223	0.154
Cakes	12.6	0.02	86.8	60.0	(69.6, 104.5)	0.507	0.765
Cheese	20.6	0.02	33.8	22.5	(28.3, 39.2)	0.835	0.312
Coffee and tea	40.1	0.02	143.4	130.6	(130.2, 156.7)	0.058	0.008
Cold Cuts	15.2	0.02	27.6	20.0	(22.6, 32.6)	0.043	0.812
Commercial juices	43.1	0.02	287.6	240.0	(263.7, 311.4)	0.012	0.000
Eggs	13.1	0.02	70.6	50.0	(57.8, 83.5)	0.050	0.373
Flavored powder	26.8	0.02	31.0	25.0	(25.0, 37.0)	0.427	0.984
Flavored Snacks	9.5	0.02	59.5	48.0	(43.2, 75.9)	0.292	0.015
Fried snacks	6.5	0.01	128.3	100.0	(96.6, 160.0)	0.347	0.618
Fruit juices	17.7	0.02	287.0	251.6	(258.9, 315.1)	0.539	0.034
Fruits	31.6	0.02	154.0	131.0	(134.1, 173.9)	0.041	0.001
Leafy Vegetables	29.4	0.02	35.7	30.0	(30.4, 40.9)	0.882	0.755
Milk	51.8	0.03	206.6	198.0	(193.2, 219.9)	0.595	0.048
Pasta	21.1	0.02	207.4	208.8	(181.7, 233.2)	0.125	0.717
Pizza	5.8	0.01	259.2	181.0	(209.6, 308.7)	0.385	0.777

Red meat	63.8	0.02	97.8	90.0	(89.3, 106.4)	0.139	0.012
Rice	87.1	0.02	144.7	125.0	(135.4, 153.9)	0.004	0.006
Salted snacks	20.1	0.02	121.0	89.9	(100.5, 141.4)	0.311	0.035
Sandwiches	3.3	0.01	200.1	228.0	.	.	.
Soft drinks	40.2	0.03	337.8	311.0	(309.7, 365.9)	0.743	0.289
Soups	2.8	0.01	383.2	390.0	.	.	.
Sugar	44.6	0.02	11.8	9.0	(9.99, 13.6)	0.506	0.011
Sweets	45.5	0.03	66.8	38.0	(53.9, 79.6)	0.052	0.000
Toast and Biscuits	34.5	0.02	77.4	50.0	(64.3, 90.5)	0.580	0.014
Tubers and roots	14.7	0.02	96.2	70.0	(73.5, 118.9)	0.043	0.809
Vegetables	33.6	0.02	63.5	50.0	(54.9, 72.0)	0.474	0.732
White meat	35.6	0.02	100.5	98.0	(90.6, 110.4)	0.136	0.267

Abbreviations: SE, Standard Error; IQR, Interquartile Range. [a] The list of foods included in each food grouping is described in Table S1. [b] Trend was estimated using logistic and linear regression models. Note: missing standard errors because of stratum with single sampling unit.

Table S3. Frequency of consumption and portion size of food groupings for adults of the ISA-Nutrition in 2003, 2008 and 2015.

	Total Adults (*n* = 1865)									
	ISA-Nutrition 2003 (*n* = 692)					ISA-Nutrition 2008 (*n* = 560)				
Food Grouping [a]	Consumption		Portion size (g)			Consumption		Portion size (g)		
	%	SE	mean	median	IQR	%	SE	mean	median	IQR
Alcoholic beverages	11.1	0.01	762.4	602.6	(610.1, 914.7)	13.2	0.02	735.6	500.6	(577.9, 893.3)
Beans	62.9	0.02	104.1	86.0	(96.9, 111.2)	66.3	0.03	108.9	86.0	(100.9, 116.9)
Breads and rolls	70.0	0.02	61.6	50.0	(58.4, 64.8)	70.4	0.02	62.1	50.0	(58.7, 65.6)
Butter and margarine	46.9	0.02	15.2	15.0	(13.3, 17.0)	40.9	0.02	16.8	15.0	(14.6, 19.0)
Cakes	10.6	0.01	91.4	60.2	(72.1, 110.7)	10.0	0.01	85.5	70.0	(71.2, 99.8)
Cheese	25.3	0.02	38.6	33.3	(34.3, 42.8)	25.8	0.02	37.6	30.0	(32.4, 42.8)
Coffee and tea	79.1	0.02	117.3	96.1	(111.0, 123.6)	75.8	0.02	115.8	96.1	(106.1, 125.5)
Cold Cuts	11.5	0.01	41.9	30.0	(34.3, 49.6)	16.5	0.02	32.5	30.0	(25.7, 39.3)
Commercial juices	17.5	0.01	306.1	257.1	(278.9, 333.3)	23.6	0.02	281.5	240.0	(258.2, 304.8)
Eggs	9.1	0.01	57.1	50.0	(52.0, 62.1)	11.6	0.02	47.6	50.0	(37.9, 57.3)
Flavored powder	9.8	0.01	31.0	25.0	(26.2, 35.8)	8.9	0.02	27.7	22.0	(20.1, 35.3)
Fried snacks	5.8	0.01	119.7	102.0	(97.4, 142.0)	5.5	0.01	151.1	130.0	(114.3, 187.9)
Fruit juices	21.0	0.02	516.9	251.6	(102.8, 931.0)	21.5	0.02	259.7	240.4	(234.4, 285.0)
Fruits	26.3	0.02	151.1	130.0	(137.0, 165.2)	34.6	0.03	172.0	140.0	(151.5, 192.5)
Leafy Vegetables	41.0	0.02	40.4	30.0	(35.3, 45.5)	43.0	0.02	43.0	32.0	(37.4, 48.6)
Milk	61.7	0.02	165.7	136.1	(155.5, 175.9)	55.7	0.03	150.2	123.8	(139.1, 161.2)
Pasta	19.6	0.02	237.6	245.0	(212.9, 262.4)	19.6	0.02	219.2	208.8	(198.4, 239.9)
Pizza	8.1	0.01	300.1	286.4	(249.7, 350.6)	8.1	0.01	209.6	200.3	(180.9, 238.2)
Red meat	58.9	0.02	117.2	100.0	(110.6, 123.7)	59.9	0.02	116.2	100.0	(104.0, 128.5)
Rice	78.1	0.02	163.4	132.5	(153.6, 173.1)	82.5	0.02	142.3	124.0	(131.5, 153.1)
Salted snacks	16.9	0.01	95.7	71.3	(78.5, 112.9)	15.6	0.02	90.3	60.0	(67.0, 113.6)
Sandwiches	4.4	0.01	234.3	202.0	(194.8, 273.8)	4.3	0.01	157.5	124.9	(106.6, 208.4)
Snacks	2.1	0.01	81.0	40.0	.	1.8	0.01	52.8	24.0	.
Soft drinks	32.7	0.02	346.5	311.4	(318.6, 374.4)	35.5	0.02	313.0	250.0	(287.9, 338.1)
Soups	9.4	0.02	431.4	319.0	(344.3, 518.6)	3.8	0.01	385.9	300.0	(256.7, 515.1)
Sugar	58.7	0.03	9.8	7.2	(8.83, 10.7)	68.6	0.03	9.4	7.8	(8.55, 10.2)
Sweets	19.7	0.02	90.3	69.7	(81.1, 99.5)	28.7	0.02	82.2	52.5	(70.5, 93.9)

Toast and Biscuits	22.6	0.02	43.4	32.0	(36.8, 50.0)	25.4	0.02	39.0	30.0	(32.1, 45.9)
Tubers and roots	13.7	0.01	112.4	90.0	(90.5, 134.3)	16.8	0.02	139.6	80.0	(81.8, 197.4)
Vegetables	45.2	0.02	78.6	60.0	(69.9, 87.4)	47.2	0.03	80.8	54.3	(63.9, 97.7)
White meat	33.6	0.02	101.7	80.0	(92.0, 111.4)	41.0	0.02	121.7	100.0	(104.5, 139.0)

Abbreviations: SE, Standard Error; IQR, Interquartile Range. [a] The list of foods included in each food grouping is described in Table S1. [b] Trend was estimated using logistic and linear regression models. Note: missing standard errors because of stratum with single sampling unit.

Table S3. (Cont.) Frequency of consumption and portion size of food groupings for adults of the ISA-Nutrition in 2003, 2008 and 2015.

	**Total Adults (n = 1865)**						
	**ISA-Nutrition 2015 (n = 613)**						
**Food Grouping [a]**	**Consumption**		**Portion size (g)**			**p [b]**	
	**%**	**SE**	**mean**	**median**	**IQR**	**trend portion**	**trend freq**
Alcoholic beverages	14.5	0.02	739.6	351.5	(530.7, 948.6)	0.853	0.113
Beans	66.1	0.03	111.2	86.0	(104.0, 118.4)	0.160	0.313
Breads and rolls	74.1	0.02	60.4	50.0	(57.4, 63.4)	0.638	0.140
Butter and margarine	48.1	0.02	17.4	15.0	(16.0, 18.7)	0.052	0.942
Cakes	13.7	0.02	94.8	70.0	(73.2, 116.4)	0.845	0.190
Cheese	25.5	0.02	37.8	30.0	(33.1, 42.5)	0.790	0.924
Coffee and tea	76.5	0.02	154.4	133.1	(140.3, 168.6)	0.000	0.276
Cold Cuts	12.9	0.01	30.7	20.0	(23.6, 37.8)	0.030	0.283
Commercial juices	28.5	0.02	296.0	240.0	(260.6, 331.3)	0.648	0.000
Eggs	12.2	0.02	74.9	50.0	(62.2, 87.6)	0.016	0.101
Flavored powder	8.5	0.01	24.2	22.0	(20.7, 27.7)	0.035	0.446
Fried snacks	4.9	0.01	103.5	100.0	(84.5, 122.4)	0.619	0.578
Fruit juices	23.4	0.02	309.3	251.6	(281.6, 337.0)	0.310	0.401
Fruits	42.7	0.02	157.2	130.0	(142.4, 172.0)	0.546	0.000
Leafy Vegetables	44.0	0.02	37.5	30.0	(33.4, 41.7)	0.504	0.384
Milk	56.9	0.03	158.7	148.5	(148.2, 169.2)	0.229	0.094
Pasta	19.8	0.02	192.9	165.0	(152.3, 233.4)	0.060	0.946
Pizza	8.5	0.01	282.1	295.2	(236.9, 327.2)	0.389	0.848
Red meat	58.2	0.02	109.3	100.0	(100.2, 118.3)	0.187	0.884
Rice	80.6	0.02	155.9	132.5	(145.7, 166.1)	0.164	0.319

Salted snacks	16.8	0.02	125.5	119.8	(107.3, 143.8)	0.039	0.924
Sandwiches	4.1	0.01	163.3	158.0	(127.8, 198.7)	0.009	0.815
Snacks	2.6	0.01	61.7	50.0	.	.	.
Soft drinks	32.6	0.02	335.1	290.0	(306.7, 363.4)	0.433	0.918
Soups	7.2	0.01	382.0	389.7	(309.3, 454.8)	0.376	0.227
Sugar	60.8	0.03	11.1	8.6	(9.86, 12.4)	0.133	0.376
Sweets	32.7	0.02	71.5	47.5	(58.7, 84.3)	0.020	0.000
Toast and Biscuits	26.3	0.02	52.2	30.0	(43.0, 61.4)	0.170	0.129
Tubers and roots	15.4	0.02	145.5	88.7	(111.3, 179.7)	0.093	0.352
Vegetables	48.5	0.03	72.8	54.6	(64.0, 81.7)	0.429	0.283
White meat	38.3	0.02	118.2	100.0	(107.7, 128.7)	0.016	0.073

Abbreviations: SE, Standard Error; IQR, Interquartile Range. [a] The list of foods included in each food grouping is described in Table S1. [b] Trend was estimated using logistic and linear regression models. Note: missing standard errors because of stratum with single sampling unit.

Table S4. Frequency of consumption and portion size of food groupings for older adults of the ISA-Nutrition in 2003, 2008 and 2015.

	**Total Older Adults (*n* = 1143)**									
	**ISA-Nutrition 2003 (*n* = 456)**					**ISA-Nutrition 2008 (*n* = 321)**				
**Food Grouping [a]**	**Consumption**		**Portion size (g)**			**Consumption**		**Portion size (g)**		
	**%**	**SE**	**mean**	**median**	**IQR**	**%**	**SE**	**mean**	**median**	**IQR**
Alcoholic beverages	7.5	0.01	401.6	301.3	(272.9, 530.3)	6.5	0.01	317.2	351.5	(243.3, 391.0)
Beans	63.0	0.02	85.6	81.9	(78.9, 92.2)	67.3	0.03	87.1	86.0	(81.1, 93.0)
Breads and rolls	74.8	0.02	50.6	50.0	(48.6, 52.6)	78.1	0.02	51.5	50.0	(48.8, 54.2)
Butter and margarine	44.2	0.02	13.2	7.5	(12.1, 14.3)	46.0	0.02	15.7	15.0	(14.2, 17.1)
Cakes	5.8	0.01	83.8	60.2	(65.9, 101.7)	9.6	0.02	73.2	60.0	(60.6, 85.7)
Cheese	30.0	0.02	37.7	30.0	(32.9, 42.4)	30.4	0.02	36.4	30.0	(31.8, 41.0)
Coffee and tea	91.7	0.01	113.2	96.1	(105.8, 120.7)	88.4	0.01	108.7	96.1	(103.6, 113.9)
Cold Cuts	7.8	0.01	31.8	30.0	(25.7, 38.0)	10.8	0.02	36.3	30.0	(25.2, 47.5)
Commercial juices	7.5	0.01	319.4	240.3	(131.2, 507.7)	15.3	0.02	225.9	240.0	(192.8, 258.9)
Eggs	8.5	0.01	54.4	50.0	(48.2, 60.6)	11.8	0.01	46.8	50.0	(38.5, 55.0)
Flavored powder	4.1	0.01	41.0	25.0	(19.2, 62.8)	4.6	0.01	19.6	12.5	(9.68, 29.5)
Fried snacks	2.8	0.01	114.5	100.0	(82.7, 146.4)	3.2	0.01	81.4	50.0	(41.4, 121.5)
Fruit juices	14.8	0.01	227.1	240.4	(206.1, 248.0)	13.9	0.02	260.0	240.3	(210.3, 309.6)

Fruits	54.4	0.03	181.4	155.0	(166.2, 196.5)	58.4	0.03	187.6	158.2	(170.2, 205.0)
Leafy Vegetables	49.4	0.02	45.7	35.0	(39.8, 51.5)	47.9	0.02	42.0	35.0	(36.7, 47.3)
Milk	72.9	0.02	156.5	140.3	(147.5, 165.5)	79.1	0.02	141.2	124.3	(133.7, 148.7)
Pasta	18.0	0.02	244.6	199.8	(187.4, 301.8)	14.9	0.02	178.6	135.0	(140.4, 216.7)
Pizza	3.1	0.01	301.1	286.5	.	2.6	0.01	167.4	113.2	.
Red meat	50.8	0.02	93.3	90.0	(87.5, 99.1)	56.8	0.02	95.2	80.0	(83.1, 107.3)
Rice	79.2	0.03	135.1	106.0	(127.3, 142.9)	80.4	0.03	126.7	112.5	(116.6, 136.8)
Salted snacks	10.5	0.02	76.6	50.0	(61.8, 91.4)	7.9	0.02	83.8	60.0	(51.0, 116.6)
Sandwiches	0.6	0.00	137.6	118.0	.	0.5	0.00	118.8	85.5	.
Snacks	0.6	0.00	41.6	50.0	.	0.6	0.00	87.7	50.0	.
Soft drinks	15.3	0.01	246.6	240.2	(214.3, 278.8)	19.4	0.02	227.5	222.2	(202.9, 252.0)
Soups	16.0	0.02	356.0	325.0	(312.0, 400.1)	15.4	0.02	383.3	325.0	(320.7, 445.8)
Sugar	54.3	0.02	9.0	7.5	(7.97, 9.95)	57.8	0.02	10.2	7.8	(8.75, 11.6)
Sweets	15.8	0.02	81.9	60.5	(65.3, 98.6)	17.4	0.02	68.8	50.0	(54.2, 83.3)
Toast and Biscuits	27.8	0.02	28.6	24.0	(26.2, 31.0)	25.6	0.02	26.0	24.0	(23.0, 28.9)
Tubers and roots	15.2	0.02	116.4	90.0	(96.7, 136.2)	16.9	0.02	105.4	80.0	(83.0, 127.7)
Vegetables	45.9	0.03	76.2	60.0	(68.4, 84.0)	50.0	0.02	82.6	50.0	(69.2, 96.0)
White meat	31.4	0.02	101.3	80.0	(90.0, 112.6)	31.8	0.02	94.5	80.0	(83.1, 105.9)

Abbreviations: SE, Standard Error; IQR, Interquartile Range. [a] The list of foods included in each food grouping is described in Table S1. [b] Trend was estimated using logistic and linear regression models. Note: missing standard errors because of stratum with single sampling unit.

Table S4. (Cont.) Frequency of consumption and portion size of food groupings for older adults of the ISA-Nutrition in 2003, 2008 and 2015.

	Total Older Adults (*n* = 1143)						
	ISA-Nutrition 2015 (*n* = 366)						
**Food Grouping** [a]	**Consumption**		**Portion size (g)**			***p*** [b]	
	**%**	**SE**	**mean**	**median**	**IQR**	**trend portion**	**trend freq**
Alcoholic beverages	10.9	0.01	498.2	304.0	(300.4, 695.0)	0.346	0.038
Beans	65.0	0.03	89.7	79.0	(83.2, 96.3)	0.374	0.646
Breads and rolls	82.8	0.02	49.2	50.0	(46.7, 51.7)	0.346	0.004
Butter and margarine	50.1	0.03	15.7	15.0	(14.5, 16.9)	0.005	0.087
Cakes	9.2	0.01	77.2	60.0	(58.7, 95.6)	0.715	0.090

Cheese	35.2	0.03	33.1	30.0	(29.9, 36.3)	0.104	0.144
Coffee and tea	92.1	0.02	123.1	101.8	(114.5, 131.6)	0.067	0.712
Cold Cuts	9.1	0.02	23.8	17.0	(19.2, 28.4)	0.019	0.575
Commercial juices	15.1	0.02	203.1	200.0	(174.9, 231.3)	0.193	0.001
Eggs	10.1	0.01	55.7	50.0	(47.7, 63.6)	0.632	0.478
Flavored powder	4.6	0.01	24.3	16.0	(16.1, 32.6)	0.177	0.746
Fried snacks	3.8	0.01	79.8	65.0	(61.1, 98.6)	0.084	0.425
Fruit juices	18.9	0.02	221.5	211.3	(195.9, 247.0)	0.559	0.079
Fruits	66.0	0.03	155.1	131.4	(143.2, 166.9)	0.003	0.002
Leafy Vegetables	51.1	0.03	37.3	30.0	(32.9, 41.8)	0.025	0.599
Milk	72.0	0.02	146.7	128.9	(136.7, 156.7)	0.201	0.622
Pasta	16.0	0.02	169.8	150.0	(145.8, 193.8)	0.020	0.500
Pizza	3.3	0.01	355.4	362.6	.	.	.
Red meat	55.0	0.02	94.2	80.0	(87.1, 101.3)	0.881	0.273
Rice	83.1	0.02	124.8	108.5	(116.3, 133.2)	0.089	0.199
Salted snacks	9.1	0.01	90.5	65.0	(60.4, 120.6)	0.420	0.600
Sandwiches	1.3	0.01	148.3	185.3	.	.	.
Snacks	0.6	0.00	17.9	10.0	.	.	0.922
Soft drinks	17.6	0.02	261.8	216.0	(228.2, 295.4)	0.424	0.339
Soups	12.4	0.02	398.3	466.0	(327.9, 468.7)	0.319	0.241
Sugar	52.0	0.03	9.4	8.0	(8.30, 10.6)	0.608	0.451
Sweets	26.7	0.03	73.7	50.0	(61.2, 86.1)	0.533	0.001
Toast and Biscuits	25.6	0.02	25.9	24.0	(22.5, 29.4)	0.227	0.504
Tubers and roots	19.7	0.02	130.2	63.9	(89.9, 170.6)	0.488	0.069
Vegetables	56.4	0.03	73.3	54.0	(62.9, 83.6)	0.557	0.010
White meat	38.2	0.03	107.1	95.0	(94.9, 119.3)	0.406	0.039

Abbreviations: SE, Standard Error; IQR, Interquartile Range. [a] The list of foods included in each food grouping is described in Table S1. [b] Trend was estimated using logistic and linear regression models. Note: missing standard errors because of stratum with single sampling unit.
